# Deficits of congenital amusia beyond pitch: Evidence from impaired categorical perception of vowels in Cantonese-speaking congenital amusics

**DOI:** 10.1371/journal.pone.0183151

**Published:** 2017-08-22

**Authors:** Caicai Zhang, Jing Shao, Xunan Huang

**Affiliations:** 1 Department of Chinese and Bilingual Studies, The Hong Kong Polytechnic University, Hong Kong SAR, China; 2 Shenzhen Institutes of Advanced Technology, Chinese Academy of Sciences, Shenzhen, China; Sun Yat-Sen University, CHINA

## Abstract

Congenital amusia is a lifelong disorder of fine-grained pitch processing in music and speech. However, it remains unclear whether amusia is a pitch-specific deficit, or whether it affects frequency/spectral processing more broadly, such as the perception of formant frequency in vowels, apart from pitch. In this study, in order to illuminate the scope of the deficits, we compared the performance of 15 Cantonese-speaking amusics and 15 matched controls on the categorical perception of sound continua in four stimulus contexts: lexical tone, pure tone, vowel, and voice onset time (VOT). Whereas lexical tone, pure tone and vowel continua rely on frequency/spectral processing, the VOT continuum depends on duration/temporal processing. We found that the amusic participants performed similarly to controls in all stimulus contexts in the identification, in terms of the across-category boundary location and boundary width. However, the amusic participants performed systematically worse than controls in discriminating stimuli in those three contexts that depended on frequency/spectral processing (lexical tone, pure tone and vowel), whereas they performed normally when discriminating duration differences (VOT). These findings suggest that the deficit of amusia is probably not pitch specific, but affects frequency/spectral processing more broadly. Furthermore, there appeared to be differences in the impairment of frequency/spectral discrimination in speech and nonspeech contexts. The amusic participants exhibited less benefit in between-category discriminations than controls in speech contexts (lexical tone and vowel), suggesting reduced *categorical perception*; on the other hand, they performed inferiorly compared to controls across the board regardless of between- and within-category discriminations in nonspeech contexts (pure tone), suggesting impaired *general auditory processing*. These differences imply that the frequency/spectral-processing deficit might be manifested differentially in speech and nonspeech contexts in amusics—it is manifested as a deficit of higher-level phonological processing in speech sounds, and as a deficit of lower-level auditory processing in nonspeech sounds.

## Introduction

Music is a universal human endowment. However, not all human beings are equally equipped with the ability to perceive or produce music. Those people with inborn musical deficits are likely to suffer “congenital amusia” (amusia hereafter), an innate neurogenetic disorder of fine-grained pitch processing from birth [1–3]. While, most studies stated that about 3–4% of the human population suffer amusia [2,4,5], a recent large-scale prevalence study reported that the prevalence of amusia might not be as high as conventionally believed, affecting approximately 1.5% of the human population [6]. It is usually believed that amusia leads to difficulties of detecting mistuned musical melodies or memorizing familiar musical melodies in affected individuals. Amusia has no obvious cause such as hearing loss, brain damage or insufficient music exposure [7,8], but it has been linked to functional and structural brain abnormalities [9–18] and probably has genetic bases [6,19].

While it has been well established that amusia primarily affects pitch processing in music, recent evidence reveals that it actually has a broader scope of influence, also affecting pitch processing in speech [20–30]. This is probably because of the important function of pitch in speech perception as well as in musical perception. For instance, linguistic information such as intonation (question/statement) and lexical tones (e.g., high level tone and high rising tone) is primarily distinguished by pitch differences [31–33]. Paralinguistic information such as emotional states (e.g., happy and sad) conveyed in speech signals is also indexed by pitch, among other acoustic cues [34,35]. It has been found that individuals with amusia exhibited inferior performance in the perception of these pitch-based linguistic and paralinguistic information [21–30]. In terms of intonation perception, several studies have found that amusics were impaired in the perception of intonation differences, especially when the pitch differences in the speech stimuli were controlled and reduced [21,25,26]. As for lexical tone perception, it has been consistently reported that amusics performed worse than musically intact controls in the identification and discrimination of lexical tones, no matter whether the amusic individuals were native tonal language speakers or non-tonal language speakers [22,24,27–30]. Furthermore, amusics were found to be less accurate than controls in identifying emotional states conveyed by prosodic differences in the speech signals [23].

Although many previous studies have pointed out that amusia is probably a domain-general deficit of pitch processing [21,24,28], it remains unclear whether this deficit is actually pitch specific or not. A few studies have reported possible impairment of amusics in segmental processing (e.g., consonants and vowels) beyond pitch processing, though the picture is far from clear [36,37]. It has been found that amusics exhibited reduced accuracy in the comprehension of news-like Mandarin spoken sentences, even when the F0 contours of the sentences were flattened to neutralize F0 information [37]. This finding suggested that amusia affects segmental processing and sentence comprehension apart from pitch processing. Another study has found that the auditory brainstem response to the complex speech sound /da/ was impoverished in the amusic brain, exhibiting reduced spectral amplitude in higher harmonic components of the auditory brainstem response, and a delayed response to the auditory stimulus [36]. This finding provides further evidence for possibly impaired processing of complex speech sounds, beyond pitch processing, in the amusic brain.

While these aforementioned findings are interesting, a full understanding of the potential deficit of segmental processing in amusics remains to be achieved. In particular, it is unclear how these findings of deficient segmental processing are related to the more fundamental and well studied deficit of pitch processing in amusics [20–30]. We speculate that the scope of deficits in amusia is broader than conventionally held, affecting frequency/spectral processing in general. Fundamental frequency (F0), the acoustic correlate of pitch, is indexed by the frequency of the first harmonic as well as the frequency distance between neighboring harmonics in the sound spectrum [38]. This means that pitch processing depends on frequency/spectral processing. Certain segments also rely on frequency/spectral analysis, such as vowels (e.g., /a/, /i/, and /u/) and sonorants (e.g., /l/ and /r/). Vowels are acoustically characterized by the frequency location of spectral peaks, or formants, in the sound spectrum [38,39]. Frequencies of the first two formants (F1-F2) are most important, which are capable of distinguishing most vowels [38,39]. The F1 frequency is generally associated with the height of a vowel, while the F2 frequency is generally associated with the frontness of a vowel. For example, a low vowel (e.g., /a/) generally has larger F1 frequencies than a high vowel (e.g., /i/), and a front vowel (e.g., /i/) generally has larger F2 frequencies than a back vowel (e.g., /u/). This means that the perception of vowels is critically dependent on the analysis and detection of the frequency location of formants in the sound spectrum. In a word, frequency/spectral processing is critical for vowel perception as well as for lexical tone perception. Thus it is likely that the inferior performance of amusics in segmental processing reported in the previous studies [36,37] is associated with an underlying deficit in frequency/spectral processing. Despite the plausibility, this hypothesis has not been systematically examined before.

To this end, we examined the performance of Cantonese-speaking amusics in vowel perception in the current study, in order to shed light on the nature of the deficit of amusia. We adopted a traditional categorical perception (CP) paradigm, which includes both identification and discrimination tasks. CP of phonemes is a fundamental property of speech perception [40,41]. CP refers to the phenomenon that two stimuli from two different categories are more detectable than two stimuli from the same category, although the acoustic difference between them is equivalent [42]. To summarize the features of CP briefly, in the identification task, there is usually an abrupt response shift across the categorical boundary; in the discrimination task, stimulus pairs which cross the categorical boundary are most discernible, whereas the accuracy of within-category discriminations is at or near chance level [43]. CP cannot be claimed if there is no advantage on the between-boundary discriminations relative to within-category discriminations.

We adopted a *group* (amusics and controls) × *stimulus type* (lexical tone, pure tone, vowel, and voice onset time (VOT)) design. The amusic participants and musically intact control participants were compared on the CP of sound continua in four stimulus contexts: lexical tone, pure tone, vowel, and VOT. While the first three types of stimuli rely on frequency/spectral processing, VOT relies on duration/temporal processing. In contrast to the deficient pitch processing that has been widely reported in amusics, duration/temporal processing has often been found to be less severely impaired or even intact in amusics [1,3,7,44]. Thus the VOT condition was used as a control condition in the current study. As for lexical tone and pure tone perception, previous studies have found that CP of lexical tones and nonspeech analogues is impaired in Mandarin-speaking amusics [22,30]. Following these findings, we predict that Cantonese-speaking amusics will also exhibit a deficit in CP of lexical tones and pure tone analogues, but they will largely preserve the ability of VOT perception. The performance of Cantonese-speaking amusics in CP of vowels is the focus of our investigation. If amusia affects frequency/spectral processing broadly, the amusic participants are expected to demonstrate inferior performance compared to the control participants in the perception of vowel stimuli, similar to their (inferior) performance in the perception of lexical tone and pure tone stimuli. Alternatively, if the deficit is pitch specific, the amusic participants are expected to show impairment only in the perception of lexical tone and pure tone stimuli, sparing the perception of vowel as well as VOT stimuli.

## Materials and methods

### Participants

15 amusic participants and 15 musically intact control participants that were matched one by one in age, gender, and years of education participated in the experiment. Another two amusic participants and one control participant who had completed the experiment were excluded from the analysis due to un-analyzable data (see Data Analysis below for details). Though no power analysis was conducted for sample size calculation, the sample size of the amusic and control participants was largely comparable to that usually reported in studies on amusia [18,24,26,45,46]. All participants were native speakers of Hong Kong Cantonese and university students in Hong Kong. They were all right-handed, with no reported history of hearing impairment, neurological illness, or long-term musical training. The amusic and control participants were determined using the Online Identification Test of Congenital Amusia (http://www.brams.org/amusia-public/?lang=en) [4]. All amusic participants scored 71% or lower, and all control participants scored above 80% in the global score of the test, which was the average of three sub-tests—out-of-key, offbeat, and mistuned—which assess musical pitch and rhythm/duration perception. Among the 15 amusic participants, 14 of them even scored below 70% in the global score. Note that the score for selecting amusics (71%) used in the current study was lower than the cut-off score for diagnosing amusics (78.4%) reported in a prevalence study on Cantonese-speaking amusics using the same test [47]. A recent study reported variation in the participants’ scores in the musical test depending on web-based experimentation or lab-based experimentation and other factors [5]. A more conservative cutoff score was used in the current study to ensure that the amusic participants were indeed impaired in musical processing. Results of independent-samples *t*-test confirmed that the global scores of the amusic participants were significantly lower than that of the control participants (*t*(28) = −13.497, *p* < 0.001). The amusic participants also performed significantly less accurately than the control participants in all three sub-tests according to the results of *t*-tests (see Table 1), but the group difference was noticeably smaller in the rhythm/duration-based sub-test (offbeat sub-test: group difference = 13.8%) than that in the two pitch-based sub-tests (out-of-key and mistuned sub-test: group difference = 29.7% and 28.8% respectively). This pattern is largely consistent with previous reports of less severely impaired or intact duration/temporal processing in amusics [1,3,7,44]. Demographic characteristics of the amusic and control participants are summarized in Table 1.

**Table 1:** Demographic characteristics of the amusic and control participants. The results (*p*-value) of independent-samples *t*-tests comparing the amusic and control participants in age and the scores of the Online Identification Test of Congenital Amusia test are also reported. n.s. = not significant.

	Amusics	Controls	*p*-value
No. of participants	15 (7 M, 8 F)	15 (7 M, 8 F)	/
Age (range)	22.1 ± 3.6 years (18.7–30.7 years)	22.1 ± 3.1 years (18.5–30.1 years)	n.s.
***Online Identification Test of Congenital Amusia***			
Out-of-key (SD; range)	62.3% (9.68; 80–47%)	91.9% (6.88; 100–80%)	*p* < 0.001
Offbeat (SD; range)	71.8% (8.14; 88–58%)	85.6% (8.36; 98–75%)	*p* < 0.001
Mistuned (SD; range)	59.3% (10.10; 79–42%)	88.1% (10.20; 100–67%)	*p* < 0.001
Global score (SD; range)	64.27% (4.65; 71–55%)	88.7% (5.23; 97–81%)	*p* < 0.001

The experimental procedures were approved by the Human Subjects Ethics Sub-committee of The Hong Kong Polytechnic University. Informed written consent was obtained from the participants in compliance with the experiment protocols. All the participants were recruited in February and March in 2016.

### Stimuli

Four types of stimulus continua were constructed: lexical tone, pure tone, vowel and VOT. Three pairs of Cantonese words, which were minimally contrastive in lexical tones, vowels and VOT respectively were selected: /ji55/ (醫 ‘doctor’) vs. /ji25/ (椅 ‘chair’) for the lexical tone continuum, /fu55/ (膚 ‘skin’) vs. /fo55/ (科 ‘section’) for the vowel continuum, and /pa55/ (疤 ‘scar’) vs. /p^h^a55/ (趴 ‘lie on one’s stomach’) for the VOT continuum. The pure tone continuum is the nonspeech analogue of the lexical tone continuum. Note that throughout the paper lexical tones are described using Chao’s tone letters [48], which are in the range of 1–5, with 5 being the highest pitch and 1 the lowest; each tone is annotated with two numbers, indicating in an abstract sense the pitch at the beginning and end of a word respectively. For instance, /55/ is a high level tone, while /25/ is a high rising tone. A male native Cantonese speaker was recorded reading aloud these three pairs of words in isolation for six times. For each pair, a clearly pronounced token was selected for generating the stimulus continuum.

**Fig 1:**
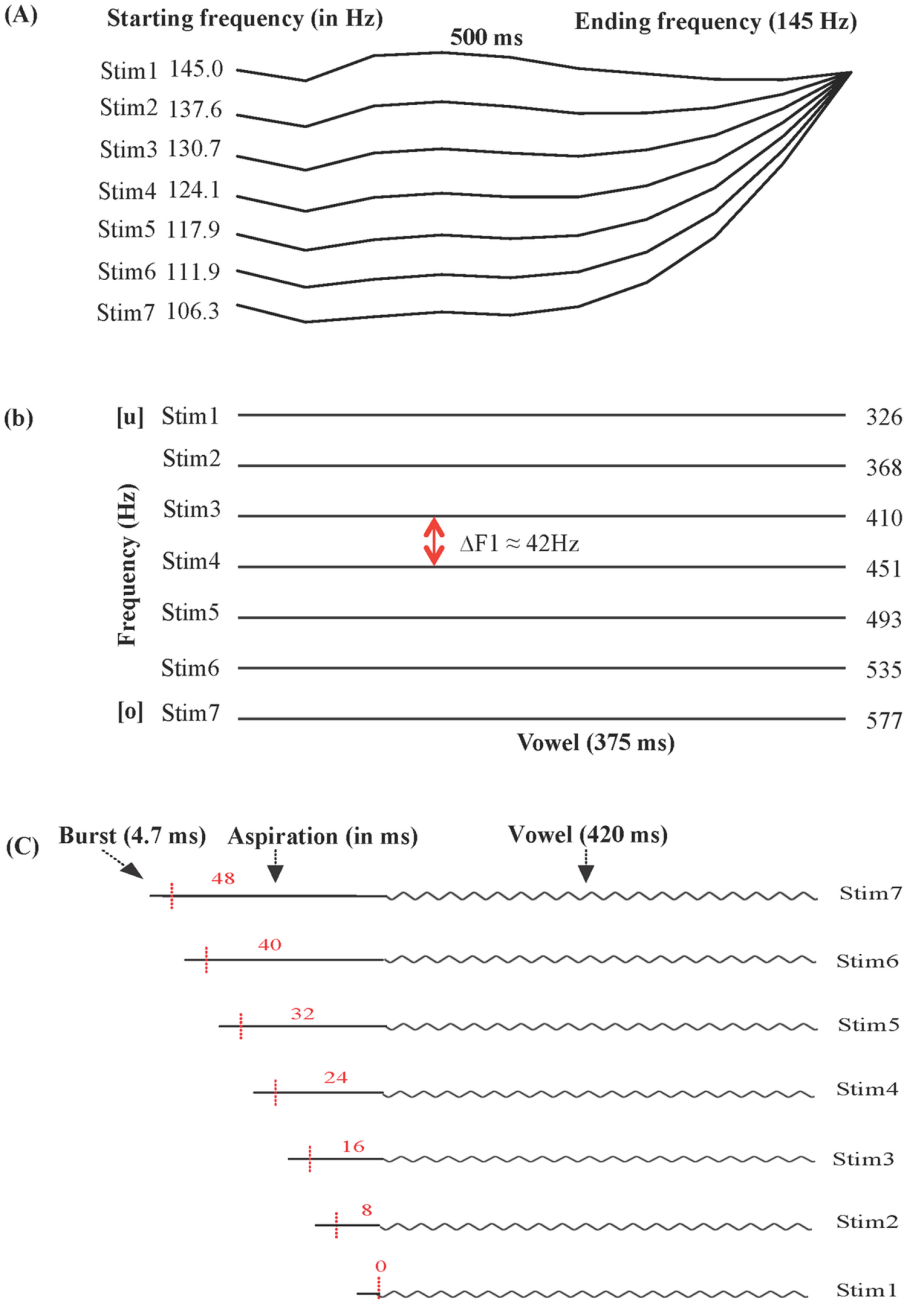
Schematic diagram of the stimulus continua. (A) Lexical tone and pure tone continuum (/55/ to /25/) with a step size of ΔF0 ≈ 0.90 semitone at the starting frequency which decreased towards the end. (B) Vowel continuum (/u/ to /o/) with a step size of ΔF1 ≈ 42Hz. (C) VOT continuum (/p/ to /p^h^/) with a step size of 8 ms.

The lexical tone continuum was created with the following procedures. First, the duration of the two selected words (/ji55/ 醫 ‘doctor’ and /ji25/ 椅 ‘chair’) was normalized to 500 ms, and their mean intensity was normalized to 60 dB using Praat [49]. Second, the F0 was measured at 11 time points at 10% intervals across the entire duration of /ji55/ and /ji25/ respectively. The F0 distance between /ji55/ and /ji25/ at each time point was then equally divided into seven steps in semitones, to derive a 7-step F0 continuum (ΔF0 ≈ 0.90 semitone at the onset of the stimuli, which decreased toward the end of the stimuli; see Fig 1A). Third, the syllable /ji55/ was used as the basis for pitch manipulation, by replacing its original F0 contour with the seven equally distanced F0 contours respectively using the overlap-add re-synthesis in Praat. In this way, a continuum of seven equally distanced pitch trajectories varying between /ji55/ and /ji25/ in semitone was generated.

The pure tone continuum were nonspeech analogues of the lexical tone continuum. First, a 500-ms pure tone sound with 15 ms rise/fall time was generated using Praat at the frequency of 145 Hz, close to the mean F0 of /ji55/. The mean intensity of the pure tone sound was 75 dB, 15 dB louder than the lexical tone stimuli, for the reason that the pure tone stimulus sounded softer. Second, the seven equally distanced F0 contours in the lexical tone continuum were extracted and superimposed on the pure tone sound, generating a continuum of seven pure tone stimuli varying in pitch between the high level and high rising tone in semitones.

The vowel continuum was created using the following procedures. First, the duration of the two selected words (/fu55/ 膚 ‘skin’ and /fo55/ 科 ‘section’) was normalized to 500 ms, and their mean intensity was normalized to 60 dB in Praat. Second, each word was segmented and divided into two parts—the consonant (/f/) and the following vowel (/u/ or /o/). The frequencies of the first to fourth formant (F1-F4) were measured at 11 time points at 10% intervals across the entire duration of the vowel /u/ and /o/ respectively. The smallest F1 value in the measurements of /u/ and the largest F1 value in the measurements of /o/ were selected as the two end points of the F1 continuum, which was then equally divided into seven steps in Hz (ΔF1 ≈ 42Hz). As for the frequencies of F2-F4, the mean frequencies of /u/ and /o/ were used, so that the frequencies were ambiguous between the two vowels. Third, using the vowel /u55/ as the basis for manipulation, seven stimuli were synthesized by setting the frequencies of F1-F4 to the designated values in seven steps using Praat. Last, the seven vowel stimuli were concatenated with the preceding consonant /f/, generating a continuum of seven equally distanced stimuli that varied in the F1 frequency between /fu55/ and /fo55/ (see Fig 1B), while the frequencies of F2-F4 were kept constant across the seven stimuli.

The VOT continuum was generated using the following procedures. First, the word /p^h^a55/, which was used as the basis for manipulation, was normalized in mean intensity to 60 dB using Praat. Second, the word /p^h^a55/ was segmented and divided into three parts: the burst release (~4.7 ms), aspiration (~48 ms), and vowel /a55/ (~420ms) (see Fig 1C). The burst release was the abrupt burst in the waveform generated by the release of the bilabial oral closure; the aspiration part covered the frication noise generated by the outward airflow following the release of oral closure; the vowel portion covered the periodic part of the waveform, and the first few periods were accompanied by some aspiration noise. The aspiration part was manipulated to vary between 0 and 48 ms in seven steps (ΔVOT = 8 ms), by shortening it proportionally using the overlap-add re-synthesis in Praat. Last, the seven lengths of the aspiration part were concatenated with the preceding burst release and the following vowel, generating a continuum of seven equally distanced stimuli that varied in VOT between /pa55/ and /p^h^a55/.

### Procedure

Each stimulus continuum was presented in an identification task and a discrimination task. In the identification task, each stimulus continuum was presented in a separate block. Within a block, the seven steps of a continuum were repeated eight times in random order, resulting in 56 randomly ordered trials (7 steps × 8 repetitions = 56 trials). The participants listened to the auditory stimuli via headphones, and were instructed to identify the heard stimulus by pressing buttons labeled with Chinese characters on a computer keyboard within 5 seconds. For the lexical tone block, the participants were asked to identify the heard stimulus as either 醫 (/ji55/ ‘doctor’) or 椅 (/ji25/ ‘chair’); for the pure tone block, the participants were informed that they would hear a nonspeech sound, and were asked to identify the heard stimulus as 醫 (/ji55/ ‘doctor’) if it resembled the tone in /ji55/, and as椅 (/ji25/ ‘chair’) if it resembled the tone in /ji25/; for the vowel block, the participants were asked to identify the heard stimulus as either膚 (/fu55/ ‘skin’) or 科 (/fo55/ ‘section’); for the VOT block, the participants were asked to identify the heard stimulus as either疤 (/pa55/ ‘scar’) or 趴 (/p^h^a55/ ‘lie on one’s stomach’).

In the discrimination task, each stimulus continuum was also presented in a separate block. A total of 18 pairs were created for each stimulus continuum, including seven identical pairs and 11 different pairs. Among the 11 different pairs, six pairs were 1-step pairs that included two stimuli separated by one step (i.e., stimulus pair 1–2, 2–3, 3–4, 4–5, 5–6, and 6–7), and the remaining five pairs were 2-step pairs that included two stimuli separated by two steps (i.e., stimulus pair 1–3, 2–4, 3–5, 4–5, and 5–7). The 2-step pairs were included in order to increase the number of trials that were relatively more perceptually distinct in a block, for the reason that the participants would otherwise get into the tendency of making ‘same’ responses all the time, which increased the chance of missing different pairs with small acoustic differences.

For each stimulus continuum, the trials were constructed by pairing two stimuli together with a 500 ms inter-stimulus interval (ISI). Note that the stimuli themselves were 500 ms in duration in all stimulus continua except the VOT continuum, where the duration of the stimuli varied between 424.7 ms and 472.7 ms. Within a block, the total number of identical and different pairs was matched. While the identical pairs were repeated eight times (56 trials), the 1-step pairs were repeated six times (36 trials) and the 2-step pairs were repeated four times (20 trials), generating a total of 112 randomly ordered pairs for each continuum. Note that half of the different pairs were presented in the forward order (AB pairs) and the other half in the reversed order (BA pairs). The participants listened to the auditory stimuli via headphones, and were instructed to discriminate whether the two stimuli were the same or different by pressing "left arrow" (same) and "right arrow" (different) on a computer keyboard within 3 seconds.

For each task, the presentation order of the four stimulus blocks was counterbalanced among the participants as much as possible. The block order was kept identical between each amusic participant and the accordingly matched control participant. The identification task preceded the discrimination task. An advantage of presenting the identification task first was that the identification task was much shorter than the discrimination task. By presenting the identification task first, it helps to ensure that the participants were not too tired when they moved on to the discrimination task after completing the identification task. Before each task, a practice block, which contained the same type of stimulus as in the first experimental block, was given to the participants to familiarize them with the procedure. In the practice identification task, the seven stimuli in a continuum were presented only once in random order. In the practice discrimination task, 15 practice trials comprising four different pairs of stimuli separated by three steps (1–4, 2–5, 3–6, 4–7) in forward and reversed orders (i.e., eight trials) and seven identical pairs (i.e., seven trials) were randomly presented.

### Data analysis

For the identification task, the probit analysis was applied to the individual identification curve of each participant to estimate the boundary position and boundary width for each stimulus continuum [50–52]. The boundary position was defined as the 50% crossover point in a continuum, and the boundary width was defined as the distance in the stimulus step between 25% and 75% of the identification responses as determined by the probit analysis [51,52]. For instance, if 25% of the stimulus 2 and 75% of the stimulus 6 were identified as /ji25/ respectively in the lexical tone continuum, the boundary width was calculated as 4 (6 − 2 = 4). The boundary width is an index of the sharpness of the response shift across the categorical boundary. The data of two amusics had to be disregarded from the analysis for the reason that no reliable boundary position can be calculated from their identification curves according to the probit analysis (e.g., the boundary position was either a negative value, or larger than the maximal stimulus step—seven). Accordingly, the data of a control participant that was matched with one of those two amusics (in terms of age, gender and years of education) had to be disregarded. The second control participant that was originally matched with the second disregarded amusics was kept in the analysis, because the second control was re-matched with another amusics in terms of age, gender and years of education. In total, the boundary position and width were calculated from 15 amusics and 15 matched controls. Two-way repeated measures ANOVAs were conducted on the boundary position and width respectively, with *group* (amusics and controls) as a between-subjects factor, and *stimulus type* (lexical tone, pure tone, vowel, and VOT) as a within-subjects factor. Greenhouse-Geisser method was used to correct the violation of sphericity where appropriate. Furthermore, in order to directly test these specific, *a priori* hypotheses that the amusic participants would perform inferiorly compared to the control participants in the lexical tone, pure tone and vowel condition, but would perform comparably in the VOT condition according to the hypothesis that amusia affects frequency/spectral processing, t-tests were conducted to compare the performance of the amusic and control participants within each stimulus type, wherever appropriate.

For the discrimination task, the data were analyzed using the sensitivity index d' [53]. The d' was computed as the z-score of the hit rate ("different" responses to different pairs) minus that of the false alarm rate ("different" responses to identical pairs) for pairs in each stimulus continuum per participant. For instance, for the pair 1–2, the hit rate was the average rate of "different" responses to different pairs 1–2 and 2–1, while the false alarm rate was the average rate of "different" responses to identical pairs 1–1 and 2–2. Based on the boundary position in each stimulus continuum for each participant obtained in the identification task, the pairs were then divided into between-category and within-category pairs for each participant, and the d' was averaged from all pairs that either spanned two categories or fell in one category for each stimulus continuum [53]. The 1-step and 2-step pairs were pooled together in the analyses of between- and within-category pairs. For instance, if the boundary position was 3.5, then 2-step pairs 2–4 and 3–5, and 1-step pair 3–4 were all deemed as between-category pairs, whereas the remaining pairs were deemed as within-category pairs. Note that the results of 1-step and 2-step pairs had also been analyzed independently, but the results were largely similar between these two step-sizes. Three-way repeated measures ANOVA were conducted on the d', with *group* (amusics and controls) as the between-subjects factor, and with *stimulus type* (lexical tone, pure tone, vowel and VOT) and *category type* (between-category and within-category) as two within-subject factors. Greenhouse-Geisser method was used to correct the violation of sphericity where appropriate. Again, in order to test the hypotheses that the amusic participants would perform inferiorly compared to the control participants in the lexical tone, pure tone and vowel condition, but would perform comparably in the VOT condition according to the major hypothesis that amusia affects frequency/spectral processing, *group* × *category type* ANOVAs were further conducted within each stimulus type, wherever appropriate.

## Results

### Identification task

**Fig 2:**
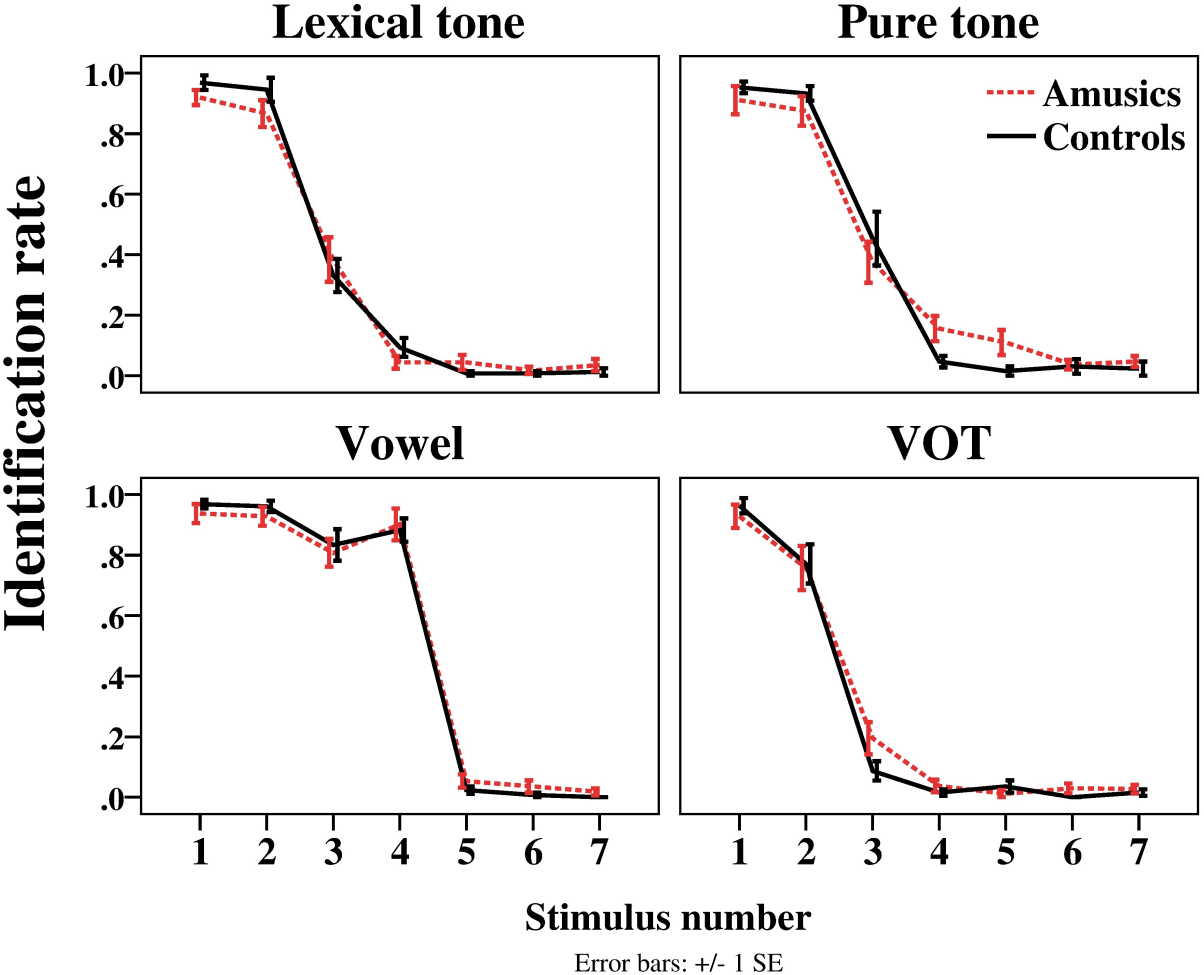
Response curves for the amusic and control participants in the four stimulus continua in the identification task. Top left panel: Rate of /ji55/ (醫 ‘doctor’) responses in the lexical tone continuum; top right panel: Rate of high level pitch responses in the pure tone continuum; bottom left panel: Rate of /fu55/ (膚 ‘skin’) responses in the vowel continuum; bottom right panel: Rate of /pa55/ (疤 ‘scar’) responses in the VOT continuum.

**Fig 3:**
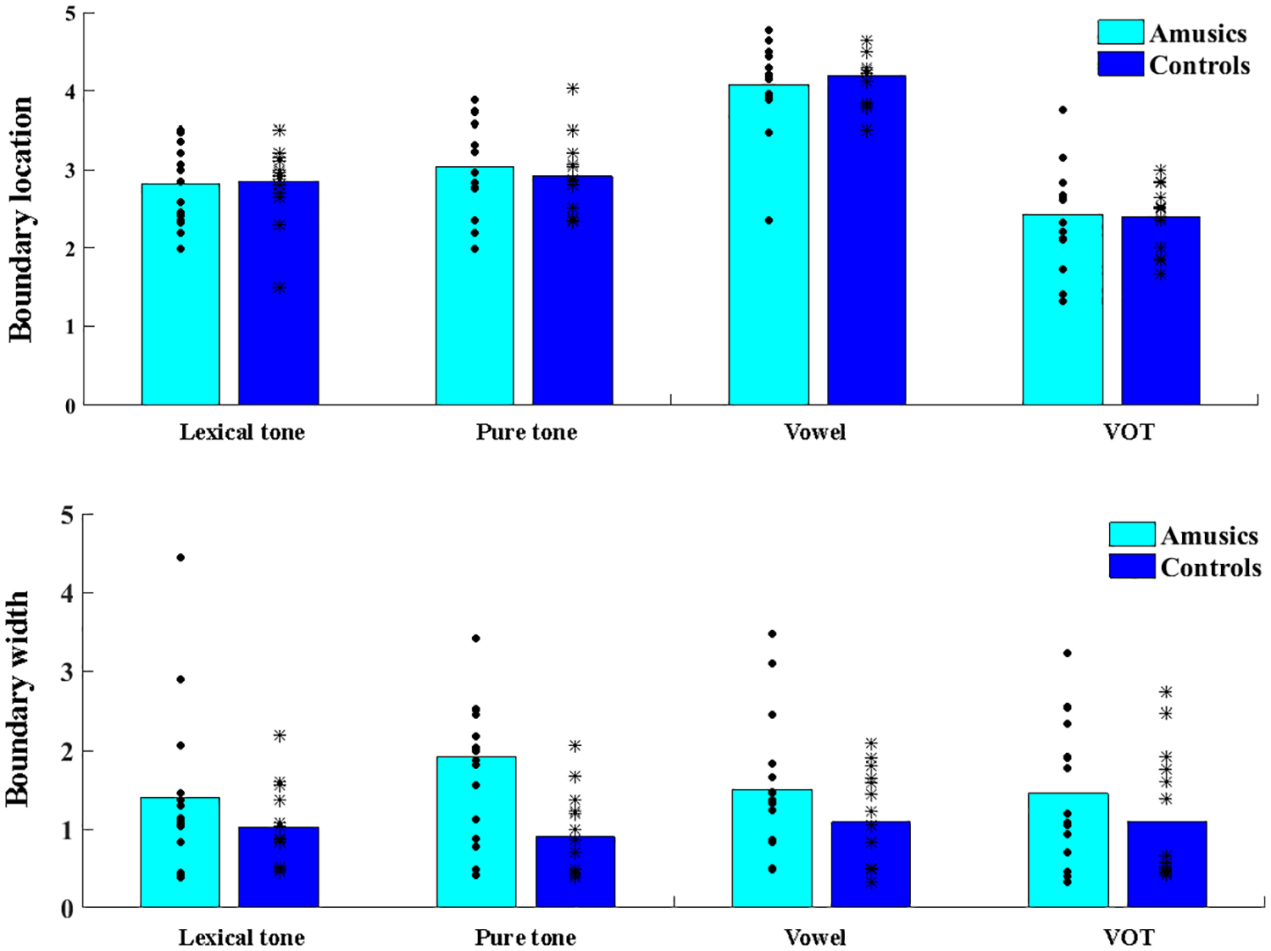
Boundary position and width for the amusic and control participants in the four stimulus continua in the identification task.

Fig 2 displays the identification curves in the four stimulus continua for the amusic and control participants, and Fig 3 shows the boundary position and width.

Regarding the boundary position, *group* × *stimulus type* ANOVA found no significant main effect of *group* or significant interaction effect of *group* by *stimulus type*. The only significant effect was the main effect of *stimulus type* (*F*(3, 84) = 61.37, *p* < 0.001, η_p_^2^ = 0.687). Post-hoc analyses revealed that the boundary position was significantly larger in the vowel continuum (*M* = 4.13, *SD* = 0.48) than in the other three conditions (*p*s < 0.001), and that the boundary position was significantly smaller in the VOT continuum (*M* = 2.41, *SD* = 0.52) than in the other conditions (*p*s < 0.05). This indicates that the response shift from /u/ to /o/ occurred later in the vowel continuum (i.e., closer to the /o/ end), and that the response shift from /pa/ to /p^h^a/ occurred earlier in the VOT continuum (i.e., closer to the /pa/ end), compared to the other continua. Regarding the boundary width, the *group* × *stimulus type* ANOVA found no significant effects. Because of the lack of significant effects of *group* and *group* by *stimulus type* interactions in the analyses of both boundary position and width, no further t-tests were conducted to examine the group difference within each stimulus type.

**Fig 4:**
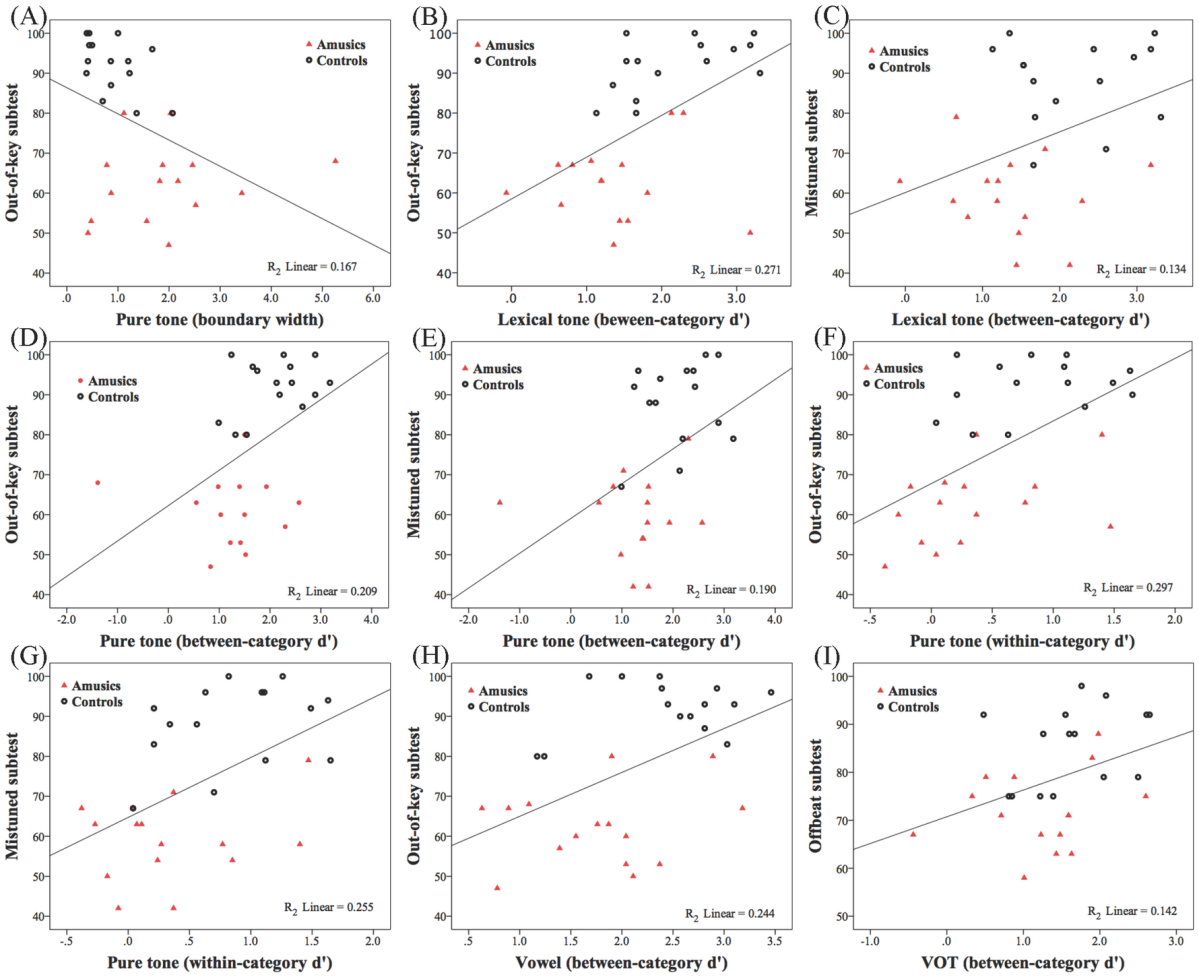
Significant correlations between the participants’ performance in the CP test and the musical scores collapsing the two groups. (A) Significant correlation between the boundary width of the pure tone condition and the score of the out-of-key sub-test; (B) significant correlation between the d' of the between-category stimuli in the lexical tone condition and the score of the out-of-key sub-test; (C) significant correlation between the d' of the between-category stimuli in the lexical tone condition and the score of the mistuned sub-test; (D) significant correlation between the d' of the between-category stimuli in the pure tone condition and the score of the out-of-key sub-test; (E) significant correlation between the d' of the between-category stimuli in the pure tone condition and the score of the mistuned sub-test; (F) significant correlation between the d' of the within-category stimuli in the pure tone condition and the score of the out-of-key sub-test; (G) significant correlation between the d' of the within-category stimuli in the pure tone condition and the score of the mistuned sub-test; (H) significant correlation between the d' of the between-category stimuli in the vowel condition and the score of the out-of-key sub-test; (I) significant correlation between the d' of the between-category stimuli in the VOT condition and the score of the offbeat sub-test.

In order to explore the relationship between the participants’ performance in the identification task and the musical test, Pearson two-tailed correlation analyses were conducted between the identification performance (boundary position and width) and the musical scores (scores in the three sub-tests and the global score). Correlation analyses were first conducted in all participants collapsing the two groups, and then within each group separately. When the two groups were collapsed, the only significant correlation was between the boundary width of the pure tone condition and the score of the out-of-key sub-test (*r* = −0.408, *p* = 0.025). This means that lower accuracy in detecting out-of-key melodies was associated with broader boundary width, namely, more gradual response shift in the perception of pure tone stimuli. The boundary width of the pure tone condition is plotted as a function of the scores of the out-of-key sub-test in Fig 4A.

Significant correlations were also found within each group, but the results were difficult to interpret, and it was not clear whether these correlations were very meaningful. Within the amusic group, positive correlations were found between the scores of the offbeat sub-test and the boundary position of the lexical tone condition (*r* = 0.576, *p* = 0.025), which seems to imply an inverse relationship between better musical duration/rhythm perception and less sensitivity towards detecting a rising pitch contour (thus larger boundary position) in the lexical tone continuum within the amusic group. Moreover, positive correlations were found between the global scores and the boundary width in the lexical tone (*r* = 0.553, *p* = 0.033) and pure tone (*r* = 0.526, *p* = 0.044) conditions. These correlations were also difficult to explain, which seemingly suggest that higher global scores were associated with more gradual response shift in the perception of lexical tone and pure tone stimuli within the amusic group. Within the control group, the global scores were positively correlated with the boundary position in the pure tone condition (*r* = 0.523, *p* = 0.045), and the scores of the out-of-key subtest were negatively correlated with the boundary position in the VOT condition (*r* = -0.697, *p* = 0.04). Future studies may further investigate the within-group correlations between musical scores and performance in the identification task.

### Discrimination task

**Fig 5:**
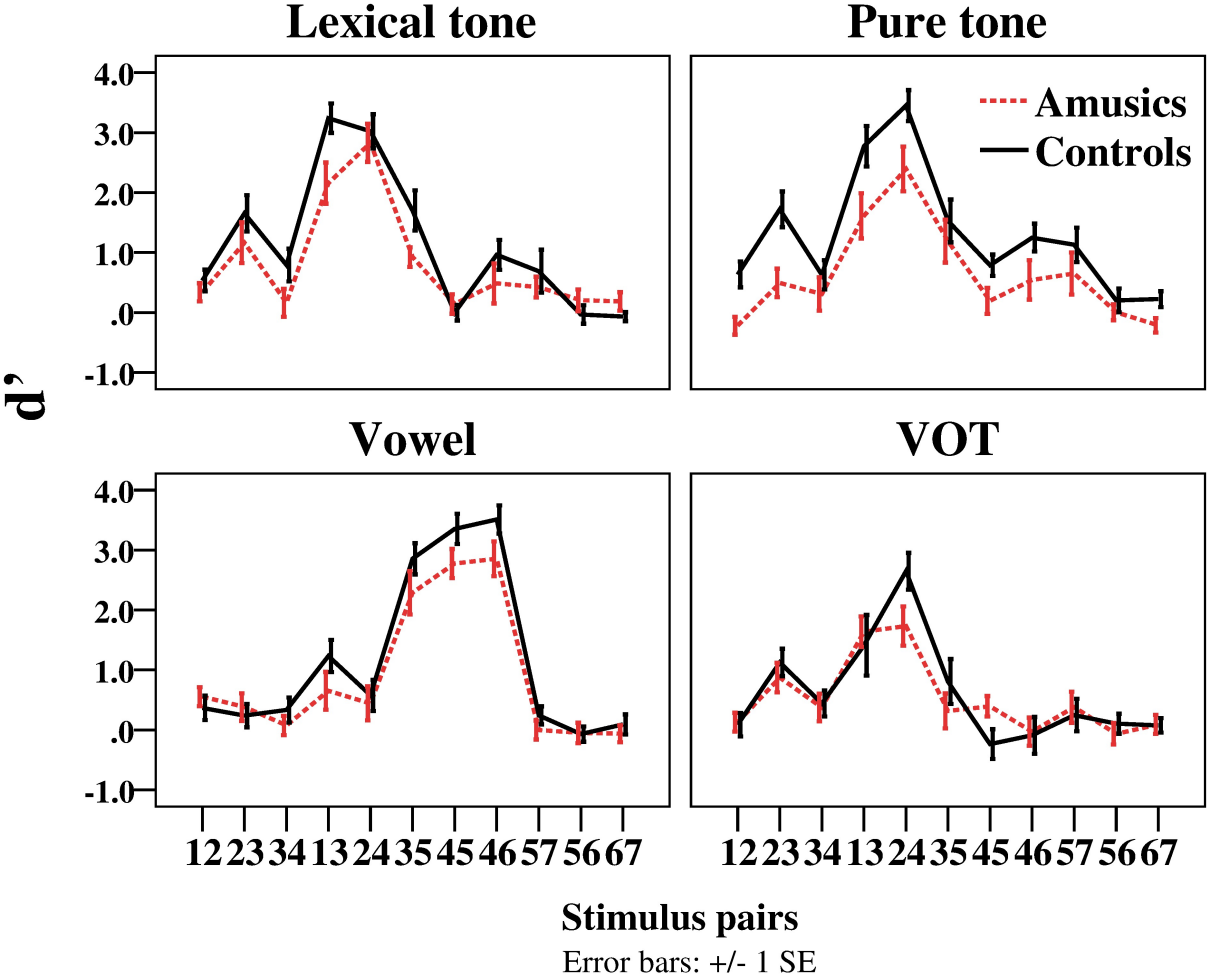
The d' of stimulus pairs for the amusic and control participants in the four stimulus continua in the discrimination task.

**Fig 6:**
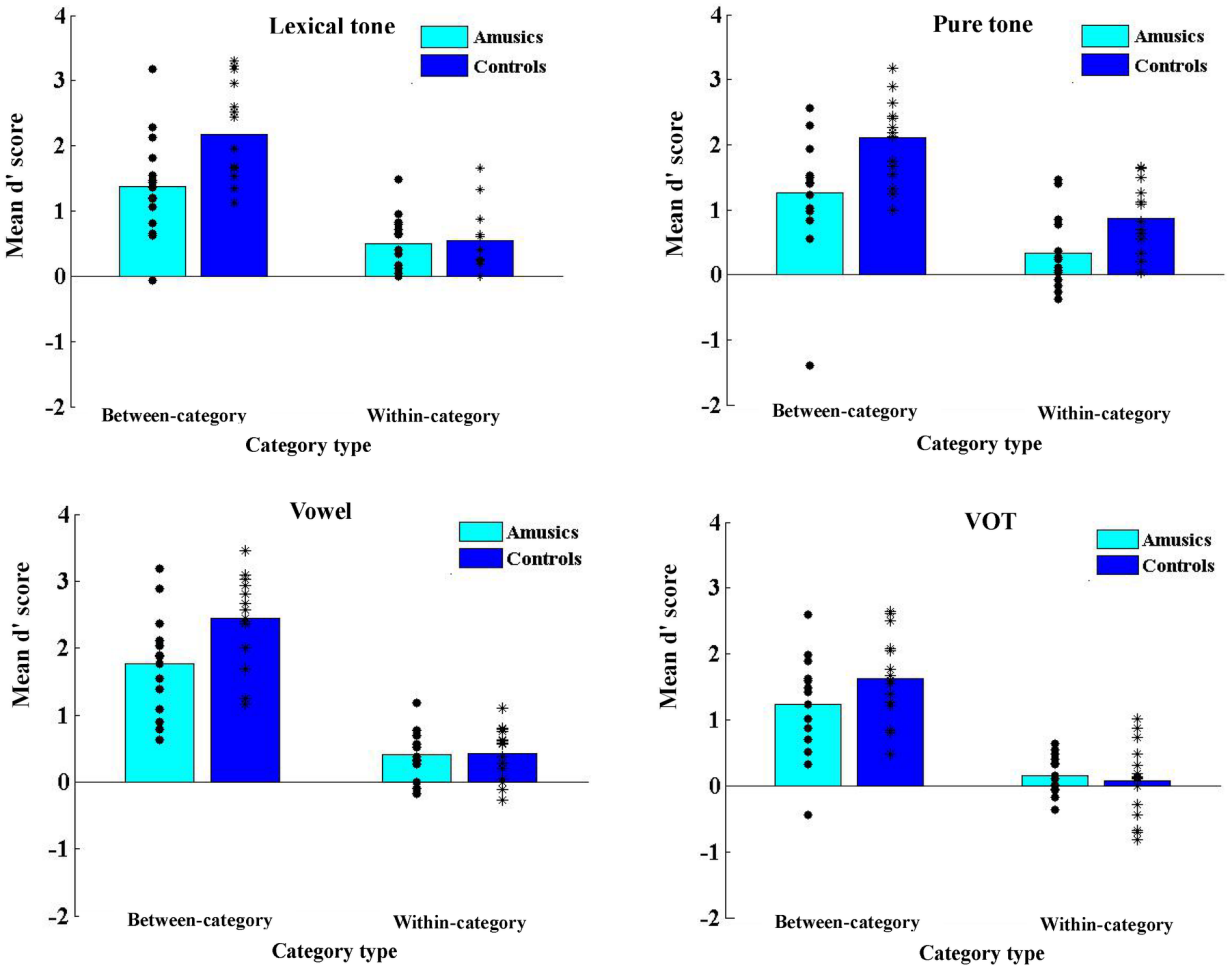
The d' of the between- and within-category pairs for the amusic and control participants in the four stimulus continua in the discrimination task.

Fig 5 illustrates the d' values for each stimulus continuum for the amusic and control participants, and Fig 6 displays the d' values averaged from between-category and within-category pairs for each stimulus continuum.

*Group* × *stimulus type* × *category type* repeated-measures ANOVA revealed significant main effects of *group* (*F*(1, 28) = 13.039, *p* = 0.001, η_p_^2^ = 0.318), *stimulus type* (*F*(3, 84) = 8.642, *p* < 0.001, η_p_^2^ = 0.236) and *category type* (*F*(1, 28) = 204.551, *p* < 0.001, η_p_^2^ = 0.880). Significant two-way interactions were found between *group* and *category type* (*F*(1, 28) = 8.931, *p* = 0.006, η_p_^2^ = 0.242), and between *stimulus type* and *category type* (*F*(3, 84) = 3.554, *p* = 0.018, η_p_^2^ = 0.113). No other effects were significant.

First, post-hoc *t*-tests were conducted to examine the interaction effect of *group* by *category type*. Results revealed that the d' was significantly lower for the amusic participants than for the control participants in between-category discriminations (*t*(118) = −4.857, *p* < 0.001, *d* = 0.887), whereas no group difference was found in within-category discriminations (*t*(118) = −1.361, *p* = 0.176). Both the amusic and control participants exhibited a benefit on the between-boundary discriminations relative to within-category discriminations, achieving higher d' scores for between-category discriminations (amusics: *t*(59) = 9.606, *p* < 0.001, *d* = 1.240; controls: *t*(59) = 15.280, *p* < 0.001, *d* = 1.973). This indicates that while both the amusics and control participants perceived the stimuli categorically, the amusic participants exhibited reduced benefit for between-category discriminations.

Second, post-hoc *t*-tests were conducted to examine the interaction effect of *stimulus type* by *category type*. Results revealed that the d' for between-category discriminations was significantly higher than that for within-category discriminations across all four stimulus types (*p*s < 0.001). For both between- and within-category pairs, there was a significant main effect of *stimulus type* (between-category: *F*(3, 116) = 3.559, *p* = 0.017, η^2^ = 0.084; within-category: *F*(3, 116) = 6.162, *p* < 0.001, η^2^ = 0.137), but pairwise comparisons with Bonferroni corrections revealed differences in the specific comparisons of stimulus types. For the between-category pairs, the d' of the VOT condition was significantly lower than that of the vowel condition (*p* = 0.010); for the within-category pairs, the d' of the VOT condition was significantly lower than that of the lexical tone and pure tone conditions (*p*s < 0.01). This means that it was most difficult to distinguish VOT differences in general (for the d' was the lowest), but there were some differences in the specific comparisons between VOT and the other three stimulus types in the between-category and within-category conditions.

In order to directly test the hypotheses of the current study, *group* × *category type* repeated-measures ANOVA was further conducted on the d' of each stimulus type, with Greenhouse-Geisser correction for the violation of sphericity. As mentioned before, the focus of this investigation is the performance of Cantonese-speaking amusics in CP of vowels. If amusia affects frequency/spectral processing broadly, the amusic participants are expected to demonstrate inferior performance compared to the controls in the perception of vowel stimuli, similar to their (inferior) performance in the perception of lexical tone and pure tone stimuli, whereas their ability to perceive the VOT stimuli would be spared. Although the three-way interaction effect was not significant in the aforementioned three-way repeated measures ANOVA analysis, there were theoretical motivations to further examine the performance of the two groups within each of the four stimulus contexts, and two-way ANOVA analysis on each stimulus context was best suited for examining these specific, *a priori* hypotheses mentioned above [54]. To this end, *group* × *category type* repeated-measures ANOVA was conducted on the d' of each of the four types of stimulus contexts.

In Fig 6, some differences between the four types of stimulus contexts can already be observed. First of all, there appeared to be a group difference in the within-category discriminations in the pure tone condition, a difference that appeared to be absent in the other three types of stimuli. Furthermore, the group difference in the between-category discriminations in the VOT condition appeared to be diminished compared to that in the other three stimulus types. These observations were largely confirmed by the results of two-way ANOVA analyses.

For the lexical tone condition, *group* × *category type* repeated-measures ANOVA found significant main effects of *group* (*F*(1, 28) = 6.421, *p* = 0.016, η_p_^2^ = 0.189) and *category type* (*F*(1, 28) = 65.367, *p* < 0.001, η_p_^2^ = 0.700), and a significant two-way interaction (*F*(1, 28) = 5.909, *p* = 0.022, η_p_^2^ = 0.174). Post-hoc analyses revealed a significant *group* effect for between-category discriminations (*t*(28) = −2.872, *p* = 0.008, *d* = 1.049), where the d' was lower for the amusic participants (*M* = 1.38, *SD* = 0.78) than for the control participants (*M* = 2.18, *SD* = 0.74). No significant group difference was found for within-category discriminations (*t*(28) = −0.284, *p* = 0.779), suggesting that the amusic and control participants performed comparably in the within-category discriminations On the other hand, the d' of between-category discriminations was consistently higher than that of within-category discriminations for both the amusic participants (*t*(14) = 3.596, *p* = 0.003, *d* = 0.928) and the control participants (*t*(14) = 8.509, *p* < 0.001, *d* = 2.197). These results indicate that the amusic participants perceived between-category lexical tone stimuli less categorically than the control participants.

For the pure tone condition, there were significant main effects of *group* (*F*(1, 28) = 10.761, *p* = 0.003, η_p_^2^ = 0.278), and *category type* (*F*(1, 28) = 63.594, *p* < 0.001, η_p_^2^ = 0.694). But the interaction effect was not significant. The main effect of *group* revealed that the d' for the amusic participants (*M* = 0.80, *SD* = 0.87) was significantly lower than that for the control participants (*M* = 1.48, *SD* = 0.86). The main effect of *category type* revealed that the d' for the within-category pairs (*M* = 0.60, *SD* = 0.60) was significantly lower than that for the between-category pairs (*M* = 1.68, *SD* = 0.89). Overall, the results indicate that the amusic participants performed worse than the control participants irrespective of between- and within-category discriminations.

For the vowel condition, there were significant main effects of *group* (*F*(1, 28) = 7.260, *p* = 0.012, η_p_^2^ = 0.206) and *category type* (*F*(1, 28) = 111.987, *p* < 0.001, η_p_^2^ = 0.800), and a significant two-way interaction (*F*(1, 28) = 4.301, *p* = 0.047, η_p_^2^ = 0.133), similar to the results of the lexical tone condition. Post-hoc analyses revealed that for the between-category discriminations, the amusic participants demonstrated significantly lower d' than the control participants (*t*(28) = -2.645, *p* = 0.013, *d* = 0.966). But the d' of the amusic participants was not significantly different from that of the control participants in the within-category discriminations (*t*(28) = −0.114, *p* = 0.910). The control participants showed greater sensitivity to the between-category pairs than to the within-category pairs (*t*(28) = 10.128, *p* < 0.001, *d* = 2.225), and similar results were found for the amusic participants (*t*(28) = 6.453, *p* < 0.001, *d* = 1.618). These results, similar to those of the lexical tone condition, indicate that the amusic participants perceived between-category vowel stimuli less categorically compared to the control participants.

For the VOT stimuli, there was only a significant main effect of *category type* (*F*(1, 28) = 78.539, *p* < 0.001, η_p_^2^ = 0.737), where the d' of the between-category discriminations was significantly higher than that of the within-category discriminations. No significant main effect of *group* or significant *group* × *category type* interaction effect was found. These results indicate that while the perception of VOT stimuli was categorical, there was no group difference in the discrimination performance.

Last, in order to explore the relationship between the perceptual performance in the discrimination task and the musical test, Pearson two-tailed correlation analyses were conducted between the discrimination performance (d' values of the between- and within-category discriminations in the four stimulus contexts) and the musical scores (scores in the three sub-tests and the global score). Correlation analyses were first conducted in all participants collapsing the two groups, and then within each group respectively. When the two groups were collapsed, in general the results revealed cross-domain or cross-task correlations between the discrimination task and musical test for frequency/spectral and duration/temporal processing respectively. Specifically, the d' values of *frequency-based* stimuli (lexical tone, pure tone and vowel) in the discrimination task were correlated with the accuracy in *pitch-based* musical sub-tests (out-of-key and/or mistuned), whereas the d' values of *duration-based* stimuli (VOT) were correlated with the accuracy in the *rhythm/duration-based* musical sub-test (offbeat). Detailed results were reported below. For the lexical tone condition, higher d' of the between-category discriminations was significantly correlated with higher accuracy in two pitch-based sub-tests (out-of-key and mistuned) and the global score (*p*s < 0.05). For the pure tone condition, higher d' in both between- and within-category discriminations was correlated with higher accuracy in two pitch-based sub-tests (out-of-key and mistuned) and the global score (*p*s < 0.05). For the vowel condition, similar to the lexical tone condition, higher d' of the between-category discriminations was significantly correlated with higher accuracy of one pitch-based sub-test (out-of-key) and the global score (*p*s < 0.05). As for the VOT condition, the only significant correlation was found between the d' of the between-category discriminations and the accuracy of the offbeat sub-test, which is a duration/rhythm-based sub-test (*r* = 0.377, *p* = 0.040). The aforementioned significant correlations are displayed in Fig 4B–4I.

Significant correlations were also found within each group, though the results were not always easy to interpret. For the amusic group, in the lexical tone condition, a negative correlation was found between the d' of the between-category discriminations and the accuracy in the rhythm/duration-based musical sub-test (offbeat) (*r* = -0.524, *p* = 0.045), which seems to suggest an inverse relationship between higher discrimination sensitivity of lexical tones and worse musical rhythm/duration perception performance. In addition, there was a positive correlation between the d' of the within-category discriminations of the lexical tone condition and the global score (*r* = 0.577, *p* = 0.024). This result was somewhat different from the correlations mentioned above with the two groups collapsed, where only the between-category discrimination sensitivity of the lexical tone condition was correlated with the global score. This likely suggests that the amusic group might be sensitive to within-category lexical tone differences, which was further correlated with their global musical scores. As for the control group, there was a positive correlation between the d' of the between-category discriminations in the lexical tone condition and the accuracy in a pitch-based sub-test (out-of-key) (*r* = 0.559, *p* = 0.03), and between the d' of the within-category discriminations in the VOT condition and the score in a duration/rhythm-based sub-test (offbeat) (*r* = 0.617, *p* = 0.014). These results were largely consistent with the correlations mentioned above with the two groups collapsed, which suggested cross-domain or cross-task correlations between speech discrimination and musical perception for frequency/spectral and duration/temporal processing respectively.

## Discussion

Congenital amusia is conventionally characterized as a disorder of fine-grained pitch processing [1–3]. However, it remains unclear whether the deficit is pitch specific or not. Previous studies implied that segmental processing beyond pitch processing is likely to be impaired in amusics [36,37]. In the present study, we hypothesized that amusia affects frequency/spectral processing. To test this hypothesis, we examined the performance of Cantonese speakers with amusia and matched musically intact controls in the CP of three types of frequency-based stimuli (lexical tone, pure tone and vowel), and a type of duration-based stimuli (VOT). The prediction was that the amusic participants would demonstrate inferior performance in the perception of vowel stimuli, and likewise in their perception of lexical tone and pure tone stimuli, but their perception of VOT stimuli would be comparable to the control participants, if there were a deficit in frequency/spectral processing. On the other hand, if the deficit were pitch specific, the amusic participants were expected to only show impairment in the perception of lexical tone and pure tone stimuli, sparing the perception of both vowel and VOT stimuli.

In the text below, we first discussed the performance of the amusic participants in the CP of frequency-based stimuli (lexical tone, pure tone and vowel) vs. duration-based stimuli (VOT) to illuminate the scope of the deficits of amusia in light of the above hypotheses. In brief, the results supported a broad deficit in the perception of frequency-based stimuli (lexical tone, pure tone and vowel) in amusia. But the data also suggested some differences in frequency/spectral processing in speech vs. nonspeech contexts. Thus we zoomed in onto the three types of frequency-based stimuli in the second section, and discussed the potentially different manifestations of the frequency/spectral processing deficit in speech vs. nonspeech contexts. In the last section, we briefly reviewed the memory mechanism of CP, and discussed the potential contribution of sensory, short-term and long-term categorical memory to the observed CP deficits in the amusic participants.

### Frequency/Spectral processing vs. duration/temporal processing

We found that in the identification task, the amusic participants performed largely comparably to musically intact control participants. The discrimination results, however, revealed a systematic deficit among the amusic participants in the processing of frequency-based stimuli. The amusic participants demonstrated worse performance than the control participants in the discrimination of all three types of *frequency-based* stimuli (lexical tone, pure tone and vowel), but showed comparable performance to the control participants in the discrimination of *duration-based* stimuli (VOT). This clear divide between frequency/spectral and duration/temporal processing was further supported by the correlation results with the two groups collapsed. While the d' of the three types of *frequency-based* stimuli (lexical tone, pure tone and vowel) was correlated with the accuracy of *pitch-based* musical sub-tests (out-of-key or mistuned sub-test or both), the d' of the *duration-based* stimuli (VOT) was correlated with the accuracy of the *rhythm/duration-based* musical sub-test (offbeat). Altogether these results suggest that the amusic participants were systematically impaired in the discrimination of *frequency-based* sound differences, while their ability of discriminating *duration-based* sound differences was less severe or largely preserved [1,7,44].

The finding of impaired vowel perception in Cantonese-speaking amusics is intriguing, and largely consistent with the previously reported results of a group of Mandarin-speaking amusics, who also exhibited impairment in vowel discrimination [55]. It was found that Mandarin-speaking amusics showed lower accuracy than controls in the discrimination of a continuum of vowel stimuli where the F1 and F2 frequencies concurrently varied between /ɤ55/ (婀 ‘fair’) and /u55/ (乌 ‘black’), but their performance in the identification of those vowel stimuli was comparable to the controls. Nonetheless, there were some refined differences between the performance of Cantonese- and Mandarin-speaking amusics, in that Cantonese-speaking amusics exhibited a deficit only in the discrimination of between-category vowel stimuli in the current study, whereas Mandarin-speaking amusics were found to perform inferiorly no matter whether the stimuli were between- or within-category pairs in the previous study [55]. While it is possible that this difference might reflect a language difference between Cantonese- and Mandarin-speaking amusics, this could also be due to the discrepancy in the stimulus design and sample size, among other factors, between these two studies. For instance, in the current study the vowel continuum varied solely in the dimension of F1 frequency, whereas in the previous study the vowel continuum varied in both F1 and F2 frequencies. It is thus possible that when the frequencies of both F1 and F2 varied, the deficit of amusia in vowel discrimination might appear to be more severe, affecting not only between-category vowel stimuli but also within-category vowel stimuli in Mandarin-speaking amusics. Furthermore, the sample size of Mandarin-speaking amusics (12 amusics) in the previous study was smaller than that of Cantonese-speaking amusics (15 amusics) in the current study, which might also contribute to the discrepancy in the results to some extent. Future studies are required to further investigate these questions, for instance, by looking into the perception of vowel stimuli that only vary in one dimension by Mandarin-speaking amusics, and also with a larger sample size of amusics.

Regardless of the small differences between Cantonese- and Mandarin-speaking amusics in within-category vowel discriminations, these findings consistently indicate that the deficit of amusia is not confined to pitch processing as conventionally held, but affects vowel perception as well. This in turn suggests that the underlying deficit of amusia is very likely to be a frequency/spectral-processing disorder instead of a purely pitch-specific disorder. It should be noted that vowels are not the only type of segments that rely on frequency/spectral processing. Certain consonants, such as sonorants (e.g., /l/ and /r/), are also cued by formant frequency differences, similar to vowels [38]. Moreover, formant transition between a consonant and the neighboring vowel (e.g., /pa/, /ta/, and /ka/) carries critical cues for the place of articulation of the consonant (e.g., bilabial, alveolar and velar) [38]. Future studies may examine the perception of other segments that are based on frequency/spectral differences, such as sonorants (e.g., /l/, /r/) and the place of articulation of stops (e.g., /p/, /t/, /k/), in order to further test the hypothesis of deficient frequency/spectral processing in amusia.

Last, the finding of impaired vowel perception in the current study could at least partly explain the previous findings of degraded performance of amusics in sentence comprehension, and impoverished brainstem response to the complex speech sound /da/ [36,37]. If those amusics were indeed impaired in frequency/spectral processing, this could lead to inferior performance in the processing of frequency-based segments, especially vowels, thereby reducing the speech comprehension accuracy and affecting the auditory brainstem response.

### Frequency/Spectral-processing deficit in speech vs. nonspeech contexts

While the amusic participants exhibited inferior performance compared to the control participants in the discrimination of all three types of frequency-based stimuli (lexical tone, pure tone and vowel), there appeared to be some differences between speech and nonspeech contexts.

In the speech context (lexical tone and vowel), both the amusic and control participants showed CP, exhibiting enhanced sensitivity for between-category discriminations relative to within-category discriminations. Importantly, however, the amusic participants performed less categorically, exhibiting less between-category benefit than the control participants, while performing comparably to the control participants in within-category discriminations. This suggests a deficit of the amusic participants in the higher-level *phonological processing* of frequency-based stimuli in speech contexts.

As for the discrimination of nonspeech stimuli (pure tone), there was a significant group difference across the board no matter whether the stimuli spanned two categories or fell within one category. It is reasonable to suggest that the perception of nonspeech stimuli primarily involved *auditory processing*. But there appeared to be some carry-over influence of long-term phonological representations of lexical tone categories from the speech domain on the perception of the nonspeech stimuli [56]. This explains why between-category discriminations were enhanced relative to within-category discriminations in the nonspeech condition, like in the speech condition. For the inferior performance of the amusic participants in the discrimination of *between-category* pure tone stimuli, multiple explanations are possible. This result could be explained by either reduced facilitatory effect of long-term phonological representations of lexical tone categories from the speech domain, a result consistent with their reduced across-category benefit in the speech condition, or impoverished auditory pitch processing ability in the amusic participants, or both. As for the inferior performance of the amusic participants in the discrimination of *within-category* pure tone stimuli, it can be primarily explained by their impoverished ability of auditory pitch processing [1–3]. Note that the control participants exhibited “un-dulled” ability of auditory pitch processing in the nonspeech context (as compared to their performance in discriminating within-category *lexical tone* stimuli), which might have further enlarged the group difference in the within-category discrimination. CP is characterized as enhanced between-category discriminations as well as dulled within-category discriminations in the speech context [56], while within-category discriminations can be dulled less in the nonspeech context [51,52,56]. As can be seen in Fig 6, the control participants exhibited higher d' in the discrimination of within-category *pure tone* stimuli than within-category *lexical tone* stimuli, a result further confirmed by the t-test (0.86 vs. 0.55, *t*(14) = -3.012, *p* = 0.009). This means that the control participants were better able to distinguish small, within-category pitch distinctions presented in nonspeech contexts than in speech contexts, an observation consistent with CP of speech stimuli [51,52,56]. Altogether, these results suggest that there was a significant group difference in auditory pitch processing of within-category pitch distinctions—while the control participants exhibited normal/un-dulled auditory pitch processing, being able to distinguish small, within-category pitch distinctions in the nonspeech context, the amusic participants exhibited impoverished auditory pitch processing of such small, within-category pitch distinctions. This result is consistent with the well-established finding that amusics are impaired in fine-grained pitch processing in nonspeech stimuli [1–3,57–59].

The above findings suggest that the frequency/spectral-processing deficit in Cantonese-speaking amusics is likely to be manifested differentially in speech and nonspeech contexts. In the speech context, the frequency/spectral-processing deficit appears to be primarily manifested as a deficit in the phonological processing of frequency-based suprasegmentals (lexical tone) and segments (vowel), whereas in the nonspeech context, it appears to be manifested in a more fundamental and profound way, affecting general auditory processing of pitch distinctions (pure tone).

The findings of impaired CP of lexical tones in Cantonese-speaking amusics are consistent with previous studies on Mandarin-speaking amusics [22,30]. It has been found that while Mandarin-speaking amusics showed a comparably abrupt response shift to the controls in the identification of two lexical tone continua (high level to high rising tone and high level to high falling tone), they failed to exhibit a robust discrimination peak across the categorical boundary in the perception of the two lexical tone continua and their nonspeech analogues [30]. This suggests that Mandarin-speaking amusics are impaired in CP of Mandarin tones, which prevails to the processing of nonspeech analogues. A recent study further confirmed that a sub-group of Mandarin-speaking amusics were impaired in CP of lexical tones, who failed to exhibit a sharp response shift across the categorical boundary in the identification as well as an enhanced peak in the discrimination [22].

Findings of the current study were largely compatible with those previous studies, showing that Cantonese-speaking amusics were impaired in CP of lexical tones. Nonetheless, there are also some small differences between the findings of the current study and the previous study [30] with regard to the amusics’ performance in nonspeech contexts. Whereas the previous study found that Mandarin-speaking amusics exhibited a deficit in CP of lexical tones in both speech and nonspeech contexts [30], results of the current study showed that Cantonese-speaking amusics were primarily impaired in higher-level phonological processing in the speech context (lexical tone), and in lower-level auditory processing in the nonspeech context (pure tone). It is not clear what caused this discrepancy, but methodological differences between the current study and the previous study might have contributed to this difference. For instance, in the current study the nonspeech stimuli were constructed from pure tone sounds, which were not very speech like. Thus the nonspeech stimuli in the current study might be more likely to reveal a deficit in auditory processing. Future studies with a more comparable design are needed to further examine the performance of Mandarin- and Cantonese-speaking amusics in CP in speech and nonspeech contexts.

No matter what, studies on CP of lexical tones in amusics have converged to show that phonological processing of lexical tones is impaired in tonal language speakers with amusia in the speech context, regardless of the specific tonal language they speak (Mandarin or Cantonese). This also means that the ability of phonological processing of lexical tones is not equally warranted in all individuals of tonal language speakers, owing to the influence of amusia and possibly other disorders too. Interestingly, the findings of impaired CP of lexical tones in amusics were similar to that of non-native speakers to some extent, for non-native speakers were also found to be unable to benefit from the between-category enhancement in the perception of lexical tones [51,52,56]. Despite the superficial resemblance, the underlying cause is likely to differ between amusics and non-native speakers. The CP deficit in amusics is presumably not due to lack of exposure to lexical tones [8], but rather because of a deficit in frequency/spectral processing. On the other hand, in (normal) non-native speakers, the CP of lexical tones could improve with increased exposure and practice.

### Sensory, short-term and long-term categorical memory

It has been proposed that multiple forms of memory—sensory, short-term, and long-term forms of categorical memory are involved in CP [56]. Sensory memory maintains the trace of a heard stimulus temporarily in memory, which is subject to rapid decay, for comparison with the sensory memory of a following stimulus [56]. Sensory memory is required for sound discriminations. While the short-term categorical representations contribute to the quasi-categorical effect sometimes observed in nonnative speakers [56], they become permanently preserved in long-term memory in native speakers via long-term language exposure. Long-term categorical representation facilitates the categorization of speech sounds in native speakers, and can also be activated during the processing of nonspeech stimuli [60,61].

A possible explanation for the impaired phonological processing of lexical tone and vowel stimuli in Cantonese-speaking amusics is that the short-term categorical representations of lexical tones and vowels might be impoverished, especially in the discrimination task. Though it is possible that amusia, a developmental disorder of fine-grained pitch/frequency processing from birth, might have some negative impact on the formation of long-term categorical representations of lexical tones and vowels in the amusics’ brain, the results of the current study are not strong enough to reach this conclusion. If the long-term categorical representations are impaired in the amusic participants, it is reasonable to expect that the amusic participants would show comprehensive impairment in the identification as well as in the between-category discrimination of lexical tone and vowel stimuli. However, the results showed that the amusic participants performed comparably to the control participants in the identification (see [30] for similar findings on Mandarin-speaking amusics). Furthermore, although the amusic participants exhibited reduced benefit in between-category discriminations than the control participants, they did perceive the lexical tone and vowel stimuli categorically, as indicated by higher d' for between-category than within-category discriminations. Altogether these results imply that the long-term categorical representation is likely to be normal or nearly normal in Cantonese-speaking amusics. Nonetheless, it should be noted that Cantonese-speaking amusics did exhibit reduced benefit across the categorical boundary for the lexical tone and vowel stimuli, which indicates that their perception was less categorical compared to controls. This may be explained by the impoverished short-term categorical representations of lexical tones and vowels in the discrimination task for sound comparison, possibly owing to the less accurate activation of long-term categorical representations into the short-term memory. Future studies may further investigate the deficit of amusics in the short-term and long-term categorical memory.

## Conclusion

To conclude, we found that Cantonese-speaking congenital amusics demonstrated systematic deficits in the discrimination of frequency-based sound distinctions, including lexical tones, pure tones and vowels, while their ability to discriminate duration differences (VOT) was largely preserved. This indicates that the deficit of amusia is not pitch specific as conventionally held, but affects frequency/spectral processing more broadly. Moreover, the frequency/spectral-processing deficit appears to be manifested differentially in speech and nonspeech contexts. The amusic participants appeared to demonstrate a deficit primarily in *phonological processing* in speech contexts (lexical tone and vowel), and a deficit primarily in *auditory pitch processing* in the nonspeech context (pure tone).

The current study has some limitations, and these issues need to be addressed in future studies. First, while the current study provided some evidence for the deficit of vowel perception in amusics, future studies should examine the perception of other segments that are based on frequency/spectral differences, such as sonorants (e.g., /l/, /r/) and the place of articulation contrast of stops (e.g., /p/, /t/, /k/), in order to further test the hypothesis of deficient frequency/spectral processing in amusics. Moreover, future studies could extend the investigation of frequency/spectral processing to amusics in non-tonal language speakers, for the current findings of impaired vowel perception in amusics are reported on tonal language speakers [55]. Second, future studies can further examine whether there are differences between Mandarin- and Cantonese-speaking amusics in CP of lexical tones in the nonspeech context. Third, the identification task was presented before the discrimination task in the current study. Although this probably did not affect the results, for the reason that the amusic and control group received the two tasks in the same order, future studies might consider counterbalancing the presentation order of the identification task and discrimination task. Last, the step size of the VOT continuum was relatively small in the current study (8 ms), which may have led to the at-chance performance in the within-category discriminations in both amusic and control groups (see Fig 6). Future studies may consider using longer VOT differences to further investigate the amusic participants’ discrimination of within-category VOT stimuli.
